# Motion Sickness, Stress and the Endocannabinoid System

**DOI:** 10.1371/journal.pone.0010752

**Published:** 2010-05-21

**Authors:** Alexander Choukèr, Ines Kaufmann, Simone Kreth, Daniela Hauer, Matthias Feuerecker, Detlef Thieme, Michael Vogeser, Manfred Thiel, Gustav Schelling

**Affiliations:** 1 Department of Anesthesiology, Ludwig-Maximilians-University, Munich, Germany; 2 Institute of Doping Analysis and Sports Biochemistry, Dresden, Germany; 3 Department of Clinical Chemistry, Ludwig-Maximilians-University, Munich, Germany; 4 Department of Anaesthesiology and Intensive Care, Medical Faculty Mannheim, University Medical Center Mannheim, University of Heidelberg, Mannheim, Germany; Universidad Europea de Madrid, Spain

## Abstract

**Background:**

A substantial number of individuals are at risk for the development of motion sickness induced nausea and vomiting (N&V) during road, air or sea travel. Motion sickness can be extremely stressful but the neurobiologic mechanisms leading to motion sickness are not clear. The endocannabinoid system (ECS) represents an important neuromodulator of stress and N&V. Inhibitory effects of the ECS on N&V are mediated by endocannabinoid-receptor activation.

**Methodology/Principal Findings:**

We studied the activity of the ECS in human volunteers (n = 21) during parabolic flight maneuvers (PFs). During PFs, microgravity conditions (<10^−2^ g) are generated for approximately 22 s which results in a profound kinetic stimulus. Blood endocannabinoids (anandamide and 2-arachidonoylglycerol, 2-AG) were measured from blood samples taken in-flight before start of the parabolic maneuvers, after 10, 20, and 30 parabolas, in-flight after termination of PFs and 24 h later. Volunteers who developed acute motion sickness (n = 7) showed significantly higher stress scores but lower endocannabinoid levels during PFs. After 20 parabolas, blood anandamide levels had dropped significantly in volunteers with motion sickness (from 0.39±0.40 to 0.22±0.25 ng/ml) but increased in participants without the condition (from 0.43±0.23 to 0.60±0.38 ng/ml) resulting in significantly higher anandamide levels in participants without motion sickness (p = 0.02). 2-AG levels in individuals with motion sickness were low and almost unchanged throughout the experiment but showed a robust increase in participants without motion sickness. Cannabinoid-receptor 1 (CB1) but not cannabinoid-receptor 2 (CB2) mRNA expression in leucocytes 4 h after the experiment was significantly lower in volunteers with motion sickness than in participants without N&V.

**Conclusions/Significance:**

These findings demonstrate that stress and motion sickness in humans are associated with impaired endocannabinoid activity. Enhancing ECS signaling may represent an alternative therapeutic strategy for motion sickness in individuals who do not respond to currently available treatments.

## Introduction

Between 7% and 28% of individuals report symptoms of acute motion sickness during road [1], air [2] or sea travel [3]. Motion sickness can be extremely debilitating and yet, the present understanding of the neurobiologic mechanisms leading to motion sickness is incomplete. The traditional sensory conflict hypothesises including the “neuronal mismatch theory” suggests that motion sickness results from a conflict between actual and anticipated signals from sensory organs sub-serving spatial orientation [4], [5]. These theories do not explain individual motion sickness susceptibility, however, and do not allow individual risk prediction for motion sickness [6]. More recent studies point to a genetic predisposition of individuals to motion sickness with a large inherited susceptibility component [7], [8]. Furthermore, motion induced nausea and vomiting (N&V) is known to be linked to a pronounced activation of the glucocorticoid- and the symphaticoadrenergic stress response systems [9]. Acute motion sickness can be regarded as the result of an intense gut-brain interaction in a stressful situation. An important regulator of this interaction under physiologic conditions and under stress is the endocannabinoid system (ECS) [10]. The ECS consists of at least two G-protein-coupled receptors named CB1 and CB2, specific endogenous ligands called endocannabinoids (e.g. anandamide and 2-arachidonoylglycerol) and a number of biosynthetic enzymes and uptake and degradation systems [11]. The ECS is assumed to connect the physical and emotional responses to stress with gastrointestinal function and energy regulation. As such, the ECS has also been regarded as a general stress recovery system [12].

There is evidence of an important inhibitory CB-receptor mediated effect of the central *and* peripheral ECS on N&V in rodents [13], [14], [15]. Furthermore, the use of the CB1-receptor blocker rimonabant in humans as an anti-obesity drug was associated with an increased incidence of N&V along with an impaired stress response [16]. This findings suggest an involvement of endocannabinoid signaling in the regulation of N&V and, presumably, also of motion sickness and stress. The role of endocannabinoids in humans with this condition has never been investigated, however. We studied the activity of the ECS in first-time participants of a parabolic flight experiment and showed that the motion sickness during kinetic stimulation was accompanied by a significantly lower reactivity of the peripheral endocannbinoid system.

## Methods

### Objectives

We studied the activity of the ECS in first-time participants of a parabolic flight experiment and tested the hypothesis whether motion sickness stress during kinetic stimulation was accompanied by changes in reactivity of the peripheral endocannabinoid system.

### Participants

Twenty-one healthy male individuals (age: 41.0±1.5 years; height: 180.6±1.6 cm; weight: 79.5±2.7 kg; body mass index: 24.3±0.6) participated in the experiments which were performed during three campaigns between May 2006 and September 2007. All subjects received full flight medical approval according to JAR-FCL 3 German version.

### Description of the Investigations and Procedures undertaken

#### Parabolic Flight

Parabolic flights (PFs) were performed with an Airbus A300 ZeroG (Novespace, France). During a parabolic flight maneuver, microgravity conditions (<10^−2^ g) are generated for approx. 22 seconds. Each parabola is initiated with a 1.8 g pull-up and terminated with a 1.8 g pull-out by the aircraft. During an average mission, 30 regular parabolas are flown with 8 min breaks (periods of horizontal flights) after 10 and 20 maneuvers. All volunteers received weight-adapted oral scopolamine (0.4–0.6 mg) 1 h before flight and were seated in a regular aircraft seat restrained by a seat belt. A possible effect of oral scopolamine on blood endocannabinoids was excluded in a ground based control experiment in 3 volunteers where endocannabinoids were measured before scopolamine, after 1 hour and after 3 hours after drug ingestion. Anandamide and 2-AG blood levels showed no significant change over time (data not shown).

#### Endocannabinoid Measurements

Venous blood samples for endocannabinoid measurements were taken in-flight before start of the parabolic maneuvers (T0), after 10 (T1), 20 (T2), and 30 (T3) parabolas, in-flight after termination of the parabolic cycles (T4) and 24 h later (T5). Blood samples were drawn into EDTA containing tubes (S-Monovette®, Sarstedt, Numbrecht, Germany), immediately frozen on dry ice and then kept at −80°C. When stored under these conditions, endocannabinoids are stable for up to 6 months [17]. The time interval between blood sampling and freezing was minimized because previous experiments have shown that endocannabinoid generation in native blood samples is continued ex-vivo [18].

Whole blood concentrations of the endocannabinoids anandamide and 2-arachidonoylglycerol (2-AG) were determined within 6 months after the experiments using a method based on high performance liquid chromatography-tandem mass spectrometry (HPLC-MS/MS) which has previously been described [18]. Our method is linear at least within a range of 0.1 to 2 ng/ml for anandamide and 0.5 to 10 ng/ml for 2-AG. The inter-assay coefficient of variation is 34% for a mean anandamide concentration of 0.2 ng/ml. The lower limit of detection of the method (defined as a signal/noise ration >4∶1) is 0.025 ng/ml for anandamide and 0.33 ng/ml for 2-AG. In biological matrices, 2-AG (including its deuterated analog) rapidly isomerizes to 1-AG [19]. We therefore quantified 2-AG as the sum of 1- and 2- esters of arachidonic acid.

#### Gene expression analyses of endocannabinoid receptors

Gene expression analysis of CB1 and CB2 receptors was performed in leucocytes isolated from blood samples (9 ml) drawn 24 h before and 4 h after the flight experiment using the LeukoLOCK™ Total RNA Isolation Kit as per manufacturer's instructions (Applied Biosystems Foster City, CA, USA). All 7 individuals who developed motion sickness were selected for gene expression analyses. Another 7 age and body weight matched participants without motion sickness served as controls. Quantity and quality of RNA was determined using a spectrophotometer; quality of the RNA revealed satisfactory in all cases (260/280 nm absorbance ratio between 1.95 and 2.15). Hereafter, equal amounts from the different samples of amplified RNA (1000 ng) were transcribed into cDNA. The reverse transcripton (RT) reaction was carried out using mixed oligo-dT and random primers and Superscript III reverse transcriptase (Invitrogen, Carlsbad, USA) following the manufacturer's protocol. Quantity and quality of the RNA was determined using a spectrophotometer; quality of the RNA was satisfactory in all cases (260/280 nm absorbance ratio between 1.95 and 2.15). Equal amounts of the different samples of RNA (1000 ng) were transcribed into cDNA. The RT reaction was carried out using mixed oligo-dT and random primers and Superscript III reverse transcriptase (Invitrogen, Carlsbad, USA), as per manufacturer's instructions.

Real-time qPCR was performed in triplicates with the Light Cycler 480 instrument (Roche Diagnostics, Mannheim, Germany). For cannabinoid receptor quantification Roche's qPCR Mastermix and highly specific fluorogenic primer-probe sets synthesized by Primerdesign, Southampton, United Kingdom, were used as previously described [20].

#### Glucocorticoid measurements

Salivary samples for cortisol measurements were obtained using Salivette® sampling devices (Sarstedt, Germany) simultaneously with blood sampling for endocannabinoid measurements at T0-T5. Free cortisol in saliva was quantified by an automated immunoassay system based on the principle of electrochemiluminescence (Elecsys Cortisol, Roche Diagnostics, Mannheim, Germany).

#### Quantification of Stress and N&V

At each time point (T0-T5), subjects completed the German *Kurzfragebogen zur aktuellen Beanspruchung* (short questionnaire of current stress; KAB [21]). The questionnaire is highly sensitive to short-term or situational changes in subjective stress experience. It is composed of 6 items of paired positive and negative adjectives, referring to perceptions of current stress and strain or relaxation (e.g. “tense–calm”, “uneasy–relaxed”). Subjects give their ratings on a six-point scale. The range for total item means is 1–6, with higher values indicating an increased stress experience. Because of the composition of the questionnaire, it is usually not possible to remember the previous ratings, thus preventing carryover effects.

Nausea scores were assessed on a verbal rating scale ranging from 1 =  no nausea to 6 =  maximal nausea at each time point. In order to document the occurrence of active vomiting, all experiments were video taped while in-flight (time points T0-T4) and analyzed offline.

### Ethical considerations

The study was approved by the Ethical Committee of the University of Munich (Protocol#152-06). All participants signed informed consent and received flight medical approval according to JAR-FCL 3 German version.

### Statistical methods

All variables were tested for normal distribution using the Kolmogorov-Smirnov test. Blood endocannabinoid and saliva cortisol concentrations at the 6 time points of measurement between volunteers with and without motion sickness were compared using a Repeated Measure General Linear Model (RM-ANOVA) with time point of measurement as a within subject effect and the occurrence of motion sickness as a between subject effect. When RM-ANOVA showed a significant effect, a t-test in normally distributed data or a Mann-Whitney U test in case of non-parametric data was used to determine at which time point endocannabinoid blood and saliva cortisol levels were significantly different. CB – receptor expression 24 h before and 4 h after the experiment was compared by paired t-test or by the Wilcoxon Signed Rank Test in case of nonparametric data. The Pearson correlation coefficient was calculated as a measure of linear association between parametric variables, Spearman's rho was used for non-normally distributed data. A p-value <0.05 was regarded as statistically significant. Data are presented as mean±SD with exception of figures, where mean±SEM is used to increase clarity. Statistical calculations were performed using PASW Statistics 17.0 and Sigma Plot 11.0, Chicago, Illinois, USA.

## Results

### Parabolic flight resulted in an acute stress reaction and motion sickness in one third of the participants

From 21 volunteers, 7 developed acute motion sickness associated N&V. From time point T1 (after 10 parabolas) to the in-flight post-parabola phase of the experiment (T4), these individuals showed significantly higher KAB stress symptom scores. Nausea scores in participants with acute motion sickness increased later than stress scores and were significantly higher between the time points T2 and T4 (Table 1).

**Table 1:** Stress and nausea during the experiment.

Score	In-flight pre-parabolas (T0)		After 10 parabolas (T1)		After 20 parabolas (T2)		After 30 parabolas (T3)		In-flight post -parabolas (T4)		24 h after parabolas (T5)	
	*Motion Sickness*		*Motion Sickness*		*Motion Sickness*		*Motion Sickness*		*Motion Sickness*		*Motion Sickness*	
	yes	no	yes	no	yes	no	yes	no	yes	no	yes	no
**KAB – Stress** a	2.4±1.2	2.3±0.9	3.4±1.4*	2.1±0.7	4.1±1.3*	1.9±0.6	3.7±1.2*	1.5±0.5	2.8±1.1*	1.7±0.7	1.5±0.6	1.7±0.5
**Nausea** b	1.0±0.0	1.0±0.0	1.1±0.4	1.0±0.0	1.9±0.7#	1.0±0.0	4.3±1.7#	1.0±0.0	2.7±1.8#	1.0±0.0	1.0±0.0	1.0±0.0

Comparison of KAB stress symptom and nausea scores between participants with and without motion sickness during the parabolic flight experiment.

*p<0.01 and

# p<0.005 when compared to participants without motion sickness. Data are mean±SD.

a German Kurzfragebogen zur aktuellen Beanspruchung (short questionnaire of current stress; KAB) [21].

b Nausea was quantified on a verbal rating scale ranging from 1 =  no nausea to 6 =  maximal nausea.

There were no significant differences regarding demographic variables between individuals who developed motion sickness and those who did not (Table 2).

**Table 2:** Demographic data.

	No motion sickness (n = 14)	Motion sickness (n = 7)
Age (y)	42.3±6.5	38.2±6.6
Size (m)	1.79±0.71	1.86±0.60
Weight (kg)	79.8±11.9	79.5±12.1
Body Mass Index	24.8±2.6	23.0±2.0

Demographic data of participants with and without motion sickness during the experiment. There were no significant differences between individuals who developed kinetosis and those who did not (p>0.11).

### Acute motion sickness was associated with significantly lower blood endocannabinoid levels

Volunteers with acute motion sickness showed significantly lower endocannabinoid blood concentrations during the exposure to kinetic stress. RM-ANOVA demonstrated a significant between group effect of motion sickness for anandamide (Type III Sum of Squares  = 0.95, F = 4.7, p = 0.04) and for 2-AG (Type III Sum of Squares  = 628.1, F = 8.4, p = 0.01).

**Figure 1:**
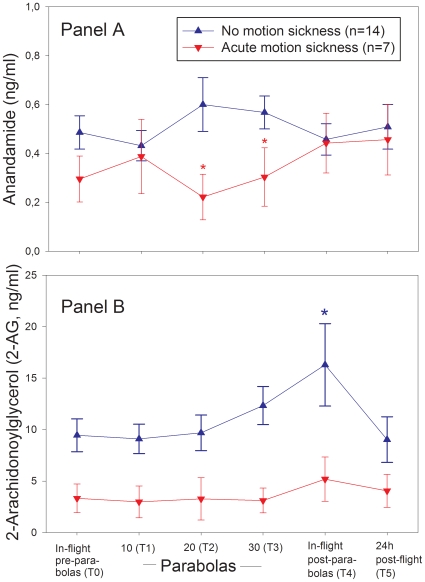
Whole blood endocannabinoid concentrations during a parabolic flight experiment in 21 male volunteers. *Panel A* compares blood concentrations of the endocannabinoid anandamide between volunteers who developed motion sickness accompanied by severe N&V (n = 7, red lines) and those who did not (n = 14, blue lines). After the 10^th^ parabolic maneuver (T1), anandamide blood concentrations dropped in individuals with motion sickness but increased in volunteers without (Mann-Whitney U Statistic  = 14.5, T = 42.5, *p = 0.02). After the 20^th^ maneuver (T2), this difference remained significant (Mann-Whitney U Statistic  = 14.0, T = 42.0, *p = 0.01). *Panel B* shows the same comparison with regard to the endocannabinoid 2-AG. 2-AG values in volunteers with motion sickness remained almost constant and non-reactive but increased in individuals without. *indicates significantly higher 2-AG blood concentrations in individuals without motion sickness after termination of parabolic maneuvers (Mann-Whitney U Statistic = 0.0, T = 85.0, p = 0.04).

After 10 parabolic maneuvers (T1), anandamide blood levels dropped in individuals with motion sickness but increased in volunteers without. After termination of parabolic cycling (T4), anandamide concentrations returned to baseline in both groups (Figure 1, Panel A). Blood concentrations of the endocannabinoid 2-AG in volunteers with motion sickness remained almost non-reactive throughout the experiment but increased continuously in individuals without motion intolerance. After termination of the parabolic maneuvers but still in flight (T4), 2-AG blood concentrations in participants who tolerated the experiment without developing motion sickness reached maximal values and were significantly higher than in volunteers with motion intolerance (Figure 1, Panel B).

Nausea scores correlated negatively with anandamide blood levels after 30 parabolas (T3, r = −0.61, p = 0.02) but not at other time points or with 2-AG concentrations. KAB stress symptom scores at T3 were negatively related to blood anandamide concentrations (r = −0.47, p = 0.04) and to 2-AG levels (r = −0.64, p<0.01) but did not correlate with endocannabinoid levels at other time points.

### Endocannabinoid receptor expression declined in volunteers with motion sickness

**Figure 2:**
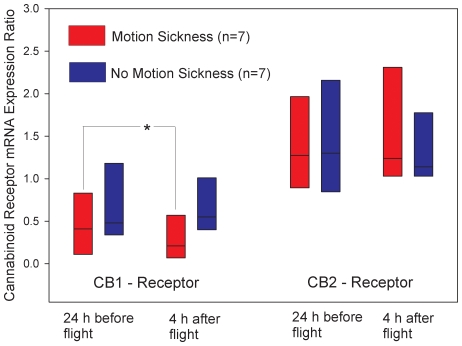
Comparison of leukocyte cannabinoid receptor mRNA between volunteers with and without motion sickness during the parabolic flight experiment. RNA was prepared from whole blood and subjected to quantitative RT-PCR (triplicate determinations). Data were calculated relative to GAPDH and RPL13A-mRNA using an efficiency-corrected algorithm. *indicates a significant decline in CB1-receptor mRNA (W = −26.0, T+ = 1.0, T- = −27.0, p = 0.03, Wilcoxon Signed Rank Test) in volunteers with motion sickness when compared to 24 h pre-flight values. CB1-receptor mRNA in individuals without motion sickness remained unchanged over time (W = −8.0, T+ = 10.0, T- = −18.0, p = 0.58) as was CB2-receptor expression before and after the experiment. Horizontal black bars in the boxplots indicate median values; upper and lower lines of the boxes show the 5^th^ and 95^th^ percentile.

Gene expression analysis was performed in leucocytes isolated 24 h before and 4 h after the parabolic flight experiment in all 7 individuals with motion sickness and in 7 matched volunteers without motion intolerance. As compared to baseline values before flight, CB1 – receptor expression at 4 h after exposure to kinetic stress declined significantly in volunteers who developed acute motion sickness but remained unchanged in participants who did not. No change over time was seen in CB2 - receptor expression (Figure 2). There was no significant difference in relative CB receptor expression at baseline 24 h before the experiment between volunteers with or without motion intolerance (CB1: 0.41 vs. 0.48, median values, p = 0.46; CB2: 1.24 vs. 1.14, p = 0.65).

### Low endocannabinoid blood concentrations were accompanied by massive activation of the HPA – axis

**Figure 3:**
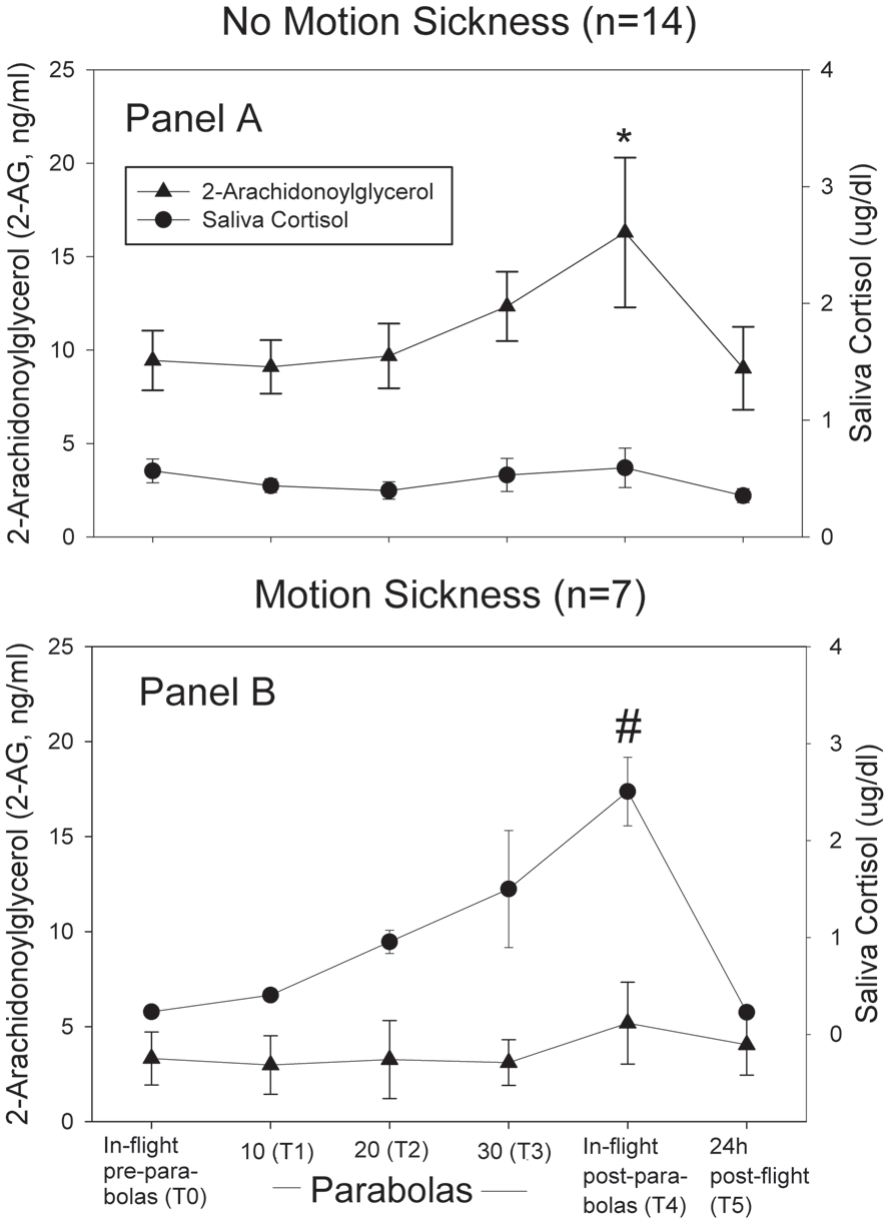
Saliva cortisol levels and 2-AG blood concentrations in volunteers with and without motion sickness. *Panel A* shows the inverse relationship between blood concentrations of the endocannabinoid 2-AG and saliva cortisol in participants without motion sickness. The increase in 2-AG during the parabolic maneuvers was associated with low saliva cortisol concentrations. *indicates significantly higher 2-AG concentrations in volunteers without motion sickness (s. Figure 2). *Panel B* illustrates the opposite association between 2-AG concentrations and saliva cortisol in volunteers with motion sickness. These individuals had low blood 2-AG levels and high saliva cortisol values. ^#^indicates significantly higher saliva cortisol concentrations in participants with motion sickness (p<0.01, t-test).

Volunteers with a low-reactive ECS and acute motion sickness showed a pronounced increase in saliva cortisol whereas cortisol values in individuals without motion intolerance remained nearly unchanged throughout the experiment (RM-ANOVA for the between group effect: Type III Sum of Squares  = 2.4, F = 4.6, p = 0.04). At the end of parabolic cycling (T4), cortisol concentrations in individuals with motion sickness were significantly higher than in motion tolerant participants (Figure 3) and were negatively related to anandamide blood levels after 30 parabolas in the complete sample (T3, r = −0.57, p = 0.03, n = 21). Saliva cortisol concentrations correlated negatively with CB1 mRNA expression 4 h after the experiment (r = −0.71, p = 0.03, n = 14) but not with CB2 receptor mRNA (r = 0.03, p = 0.96, n = 13). Positive relationships were seen between saliva cortisol at T4 and KAB stress scores (r = 0.83, p<0.01, n = 21) as well as nausea intensity (r = 0.72, p<0.01, n = 21).

## Discussion

The results of this study point to the fact that the ECS is involved in the neurobiologic mechanism of motion sickness. Previous studies have already suggested that the ECS has an important role in the pathophysiology of N&V induced by diseases (e.g. migraine) [22] and many drugs (e.g. cancer chemotherapy) [13], [23]. In animal experiments, cisplatin induced emesis could be blocked by cannabinoid agonists [14]. This anti-emetic effect of cannabinoids involves both central and peripheral mechanisms [24] and is mediated by both CB1- [14] and CB2-receptors [25], [26]. Emesis and emetic afferences from the gut are controlled by the dorsal vagal complex of the brainstem which consists of the area postrema, the nucleus tractus solitarius and the dorsal motor nucleus of the vagus. There is strong evidence supporting the presence of CB1- and CB2-receptors in these brain areas mediating anti-emetic responses [15], [26], [27]. Although measured peripherally, individuals from our study with acute motion sickness showed both, reduced blood endocannabinoid levels and a downregulation of CB1-receptor expression. A peripheral site of action of endocannabinoids at the terminals of vagal afferents in the gastrointestinal tract has recently been demonstrated where endocannabinoids are involved in the complex regulation of food intake [28] as well as in a downregulation of toxin induced emesis [29].

Our finding of differences in CB1-receptor expression between individuals with and without motion sickness is more difficult to explain, however. While blood endocannabinoid levels could reflect to some extent a spill-over of endocannabinoids from peripheral tissues involved in N&V as well as cortisol release [30], this does not apply to CB1-receptors which were measured on blood cells. Blood cells themselves are, however, a significant source of endocannabinoids [18] and this, together with the CB1-receptor expression measured on these cells indicates differences in stress-induced activation of the ECS in blood cells between individuals with and without motion sickness. An activation of the peripheral ECS by physical [31] and emotional stress [32] has recently been demonstrated. Within this context it is of interest to note that volunteers who later developed motion sickness had, albeit not significantly, lower endocannabinoid blood levels in-flight during the pre-parabola phase of the experiment when kinetic stimulation was low but stress exposure may have been already high. This suggests differences in the endocannabinoid response to stress between individuals prone to develop motion sickness and those who are not. The observation that lower endocannabinoid levels in participants with motion sickness were accompanied by a lower expression of CB1-receptors may appear counterintuitive but could point to a failure of upregulation in endocannabinoid signaling during stress exposure. There is also preliminary evidence that alterations of the ECS in blood cells mirrors dysfunctions of central endocannabinoid signaling and that peripheral blood may serve as a reservoir of anandamide for the brain [33]. Thus, lower peripheral endocannabinoid activity in individuals with motion sickness may indicate central ECS dysfunction which could help to explain many central symptoms of motion sickness including N&V [34].

Another interesting observation from our study is that anandamide and 2-AG seem to play different roles in the adaptation to parabolic flight stress and motion sickness. Changes in anandamide blood levels are seen earlier and seem more associated with nausea and the stress reaction *during* the parabolic maneuvers, whereas changes in 2-AG blood levels appear later and seem more closely related to stress recovery immediately after the parabolas. 2-AG is known to be more selective for CB1 receptors which play an important role in stress recovery reactions [35].

Participants with acute motion sickness did not only show low endocannabinoid signaling, they also demonstrated a massive activation of the hypothalamic-pituitary-adrenal (HPA) axis. Although one could assume that HPA-axis activation under the conditions of acute motion sickness is simply a non-specific reaction to stress, there is increasing evidence that endocannabinoids provide a tonic and highly specific feedback to control HPA-axis activity [36]. These effects may occur both at a peripheral [37] as well as on a central level where it has been shown that the ECS is a negative regulator of the HPA-axis and that the levels of 2-AG (but not anandamide) in the hypothalamus regulate the corticosterone response to stress [10]. In particular, high levels of 2-AG dampen HPA-axis activity whereas a stress associated decline in 2-AG levels reduced activation of CB1-receptors resulting in increased HPA-axis activity [38]. These findings from animal experiments remarkably resemble our observations in humans and give further evidence that a failure to up-regulate and to maintain endocannabinoid signaling during kinetic stimulation may result in both, an increased risk for the development of motion sickness with N&V and a pronounced stress response. An impairment in endocannabinoid signaling may therefore be an important link between stress responsiveness and the development of motion sickness and could represent a neurobiologic mechanism leading to this common disorder. Our findings also suggest that pharmacologic enhancement of endocannabinoid signaling may represent an alternative prophylactic or therapeutic approach for motion sickness in patients who do not respond to currently available treatments [13], [39].

### Limitations

This study investigated only a limited number of subjects and a larger sample size would have been preferable. However, due to the severity of the model the limited sample size appears to be less critical as the subjects' stress responses with or without motion sickness were clearly distinguishable. Other limitations include possible effects of the antiemetic drug scopolamine on our findings. Although all participants received the compound and the drug did not affect endocannabinoid blood levels directly, this does not rule out that muscarinic receptor blockade could influence the sensitivity of CB-receptors. Interactions between muscarinic receptors and endocannabinoid signaling have been observed in the hippocampus, a brain area critical for spatial orientation [40]. Although such an interaction cannot be ruled out completely, the effect should at least be the same in both groups. Performing the experiment without anti-emetic medication would be difficult to justify.

Our study did not address the question if lower endocannabinoid blood levels in individuals with motion sickness were due to baseline differences in the expression of enzymes involved in endocannabinoid biosynthesising or degradation [41]. If no such difference exists, this would suggest variations in transcriptional control events over endocannabinoid signaling. These important questions will have to be addressed in further studies.
